# Reading and writing difficulties and self-rated health among Danish adolescents: cross-sectional study from the FOCA cohort

**DOI:** 10.1186/s12889-019-6931-x

**Published:** 2019-05-10

**Authors:** Mette-Marie Zacher Kjeldsen, Christina Malmose Stapelfeldt, Louise Lindholdt, Thomas Lund, Merete Labriola

**Affiliations:** 1 DEFACTUM, Central Denmark Region, P. P. Oerums Gade 11, 8000 Aarhus C, Denmark; 2 Department of Public Health, University of Aarhus, Bartholins Allé 2, 8000 Aarhus C, Denmark; 3 Center for Social Medicine, Frederiksberg and Bispebjerg Hospital, Nordre Fasanvej 57, 2000 Frederiksberg, Copenhagen, Denmark; 4 Research Centre for Youth and Employment, Regional Hospital West Jutland, University Research Clinic, Herning, Denmark; 5 Department of Public Health, University of Copenhagen, Oester Farimagsgade 5, 1014 Copenhagen K, Denmark

**Keywords:** Reading and writing difficulties, Self-rated health, Adolescent, Public health

## Abstract

**Background:**

People struggling with reading and writing difficulties may have poor odds of achieving a good and healthy life. Reading and writing difficulties are independent risk factors for not completing education and unemployment, which are essential in order to obtain a good and healthy life. Therefore, the purpose of this study was to investigate the association between reading and writing difficulties and self-rated health among adolescents, and to investigate how mental health mediates the association.

**Methods:**

A cross-sectional study was performed based on the FOCA cohort, a Danish population-based survey among 9th grade pupils, mainly aged 15- and 16-years old, gathered during the first months of 2017. The study population contained 9748 pupils. The dependent variable was a *yes-or-no* answer to experiencing limitations in every-day life due to reading and writing difficulties. The independent variable was measured with the SF-36 self-rated health question, dichotomised in *high* (very good, excellent) and *low* (good, fair, poor). A logistic regression model was applied.

**Results:**

Among the study population 953 (9.8%) pupils reported having reading and writing difficulties. The adjusted OR of having a low self-rated health was significantly higher among adolescents with reading and writing difficulties than without (1.37 (95% CI: 1.14–1.66)). Loneliness and perceived stress, explained a minor part of the association, OR attenuated from 1.77 (95% CI: 1.51–2.09) in the crude model to 1.47 (95% CI: 1.23–1.74) in a more adjusted model.

**Conclusion:**

Adolescents with reading and writing difficulties are not only struggling with reading and writing difficulties but experiencing also low self-rated health. Mental health only explained a minor part of this association. To clarify whether causal relationship between reading and writing difficulties and self-rated health may exist, longitudinal studies are needed. If support for the hypothesised causality is found early identification of reading and writing difficulties is important, to prevent future health inequality in adolescents with reading and writing difficulties.

**Electronic supplementary material:**

The online version of this article (10.1186/s12889-019-6931-x) contains supplementary material, which is available to authorized users.

## Background

Sound reading and writing skills are essential factors in order to obtain labour market success [1, 2] and thereby a good and healthy life [3]. Thus, already in childhood and adolescence, the possibility of achieving a successful and healthy life is being founded [4]. Reading and writing skills may open up opportunities in life which may be the foundation of a good health presently and in the future as adolescents and adults [1]. Assessing overall perception of adolescents’ own health status, is commonly done using a single item asking about their self-rated health [5, 6], and studies have shown that it strongly predicts outcomes such as morbidity and mortality [7, 8].

Overall, boys tend to have a slightly better self-rated health than girls [7, 9]. For both genders in adolescence, self-rated health seems to a large extent to be based upon mental health [10], which may explain the negative association between perceived stress in adolescence and mental- and physical health [11]. Studies also point to the fact that loneliness through adolescence increases the risk for poor self-rated health, psychological and physical health in adolescence and into adulthood [12, 13]. Besides, self-rated health has also been associated with physical, personal and behavioural factors [9]; for instance a poor self-rated health among adolescents has shown to be predictive of different markers of biological dysregulation in early adulthood [14]. Socioeconomic status (SES) seems also to be predictive of self-rated health among adolescents, and negative life events during childhood have been shown to be predictive of different depressive problems [7, 9, 15, 16]. Another essential factor affecting adult self-rated health is education, thus low education increases the risk of low self-rated health [17, 18]. Education is in most cases a prerequisite for future employment and income, which are some of the basic factors in a good and healthy life [3, 19, 20]. For that reason, education is a social determinant of health, and low education contributes to increased inequality in health [4].

Reading and writing difficulties (RWD) may lead to low education, and thereby low income in adulthood [1, 2, 21, 22]. RWD cover a wide spectrum of different difficulties whereas dyslexia is the predominant cause [23]. It is estimated that between 5 and 22.5% of children and adolescents suffer from RWD, depending on the population under investigation [24–26]. In a Danish context the Danish Health Authority estimates the prevalence of dyslexia to be between 7 and 20% [27, 28], whereas the prevalence of RWD is expected to be even higher. Besides being strongly correlated with educational attainment, RWD are also associated with a number of negative factors, such as ADHD [29], criminal conduct [30] and an increased risk of internalizing, anxiety and depressive symptoms [31].

Based on these negative associations, it seems plausible that RWD independently may be associated with poor self-rated health. If in fact the association between RWD and self-rated health is already present in adolescence, this is an inequality in itself, and it reaches far beyond health inequality in adolescence.

Functional literacy and numeracy have been shown to be associated with self-assessed health in an adult population [32]. In addition, a pattern with poor health-related outcomes among adults having RWD was shown [31, 33–37], although one study did not find this association to be statistically significant [37]. Amongst adults, it has been shown that different types of RWD are associated with health-related quality-of-life (HRQoL) [38, 39]. As with adults, it seems that RWD among children and adolescents are risk factors for different emotional and mental problems such as low self-esteem, anxiety and unhappiness [31, 40]. Arkkila et al. found poor mental outcomes to be more pronounced among adolescents with specific language impairment compared to adolescents without it. Despite that, they found no difference in HRQoL between the two groups when measuring with the 16D-questionnaire validated for use among adolescents [41]. However, several other studies found that poor mental health status explained differences in HRQoL none of them being measured with the SF-36 instrument, though [42–45]. The current scientific literature does show a pattern where RWD is associated with poor health-related outcomes, even though no studies to our knowledge have investigated the relationship between RWD and self-rated health measured by the SF-36 among adolescents.

Thus, the overall purpose of this study was to investigate the association between RWD and self-rated health among adolescents. Since adolescents to a high degree may base their self-rated health on their mental health, the purpose was moreover to investigate how loneliness and perceived stress, as indicators for mental health, mediate the association between RWD and self-rated health, thereby identifying significant possible risk factors for future prospective studies.

## Methods

### Design

In this cross-sectional study, questionnaire data was gathered from a Danish population-based survey during the first months of 2017, forming the Future Occupation of Children and Adolescents cohort (the FOCA cohort) survey, [46, 47]. The questionnaire was not developed particular for this study.

### Population

#### The FOCA cohort

The FOCA cohort questionnaire was sent out as an online survey to all Danish public, private, continuation and special schools, having 9th grade pupils. The questionnaire contained questions about how the adolescents experienced different aspects of their life, such as family, friends, and spare time, in order to be able to investigate youth, health and work in future studies applying a life-course perspective.

The Danish school system has 10 years of compulsory schooling from the age of approximately 5 until the age of 15 or 16 years. By the age of 15 or 16 years, the pupils are most likely attending the graduating class, the 9th grade. Therefore, the source population was all 9th grade pupils in Denmark in the spring of 2017.

The schools were considered a unit for the data collection of the FOCA cohort, where an extraction of schools from the National Agency for IT and Learning at the Ministry of Education formed the basis for the identification of potential schools. A total of 2618 schools were identified, where 1746 schools deemed to be eligible after a complete screening of the schools. The eligible schools were contacted and invited to participate, whereof 650 schools agreed to participate and were included in the final cohort representing a total of 13,100 adolescents from all school types in Denmark [46].

The sampling within the schools and the content of the questionnaire is described in detail elsewhere [46].

#### Study population based on the FOCA cohort

**Fig. 1:**
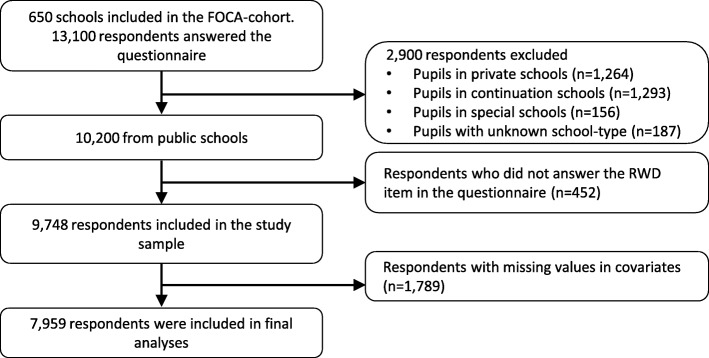
Flowchart of the selection of participants

The inclusion criterion for this particular study was being 9th grade pupils in a public school. The choice of adolescents from public schools was to get as homogeneous a group as possible. Adolescents from special schools, private schools and continuation schools were excluded, as the pupil composition can stand out either in the power of pupils with special needs (special schools), or pupils from abroad, who are only there in shorter time (private schools and continuation schools), thus 10,200 respondents from the FOCA cohort were eligible for this particular study. Pupils that did not respond to the item: “Do you feel limited by reading- or writing difficulties in your everyday life?” as an indicator for RWD were excluded (*n* = 452). Leading to a final study sample consisting of 9748 respondents (95.6%, Fig. 1).

### Data

#### Dependent variable

Information on self-rated health was based on the item: “In general, would you say your health is”; with response categories; *poor*, *fair*, *good*, *very good* and *excellent*. The item originates from the SF-36 questionnaire on health-related quality of life [48]. Both the original SF-36 and the Danish version were validated for the age of 14 and older [48, 49]. The item was dichotomised into low (*poor, fair, good)* and high (*very good, excellent)*, low being the reference group [50].

#### Independent variable

RWD was present/absent when the question: “Do you feel limited by reading- or writing difficulties in your everyday life?” was answered by *yes* or *no* (reference group), respectively. The question was formulated and used in another Danish youth cohort questionnaire survey, the West Jutland Cohort Study [51]. The item was modified for the FOCA cohort questionnaire [47].

#### Potential confounders

Gender and age were obtained from the Danish Central Person Register (CPR).

Self-assessed SES was measured by a 10-step cantril ladder. The respondents were asked to imagine the Danish society as this ladder, letting 10 represent those who are best off, and 1 represent those who are worst off. The respondents were then asked to mark the step that represented their family’s position, with higher steps on the ladder indicating higher SES. The item originates from the MacArthur Scale of Subjective Social Status and was validated for use among adolescents [52]. The variable was categorised into three groups: low (steps 1–3), medium (steps 4–7) and high (steps 8–10) [16].

Information on negative childhood events stemmed from the Norwegian Young HUNT 3 questionnaire (9 questions), 1 question from the West Jutland Cohort Study and 1 question was specially formulated for the FOCA cohort [47]. The respondents were asked if any of the following events have happened to them during their life; illness of a family member, death of a loved one, a catastrophe, a serious accident, have experienced or observed violence, have been sexually uncomfortable with either someone their own age or an adult, been threatened by other students over a longer period, been bullied, that their parents divorced. Response categories were *No*; *Yes, last year*; *Yes, in my life.* According to a former study the items were scored *no* and *yes*, respectively [53]. A missing answer were scored as *no* if the respondent had answered at least one of the other questions concerning negative childhood events [15]. The item was categorised as; *0 events*, *1–3 events*, *4–7 events* and *8–11 events*.

Loneliness was from The Three-Item Loneliness Scale, which is validated for use in large surveys [54]: “How often do you feel that you lack companionship?”, “How often do you feel left out?” and “How often do you feel isolated from others?”, with response categories *hardly ever (1)*, *some of the time (2)* and *often (3)*. The items are not validated in a Danish context. The sum scale ranges between 3 and 9 [55] with high scores representing a high degree of loneliness. The scale became missing if the respondent did not answer all three questions. The scale was dichotomised in *not lonely (1–5)* and *lonely (6–9)* [56, 57].

The 10-item Perceived Stress Scale, which measures the degree to which situations in one’s life are perceived as stressful [58] was used: “In the last month, how often..?” followed by 10 statements with a series of rating scales with response options ranging from *never* to *very often*. The measure is validated for use in a Danish context [59], and each item was scored 0–4, giving a sum scale ranging from 0 to 40 with high scores indicative of stress. The scale became missing if the respondent did not answer all the questions. The distribution of the scale allowed for using it as a continuous variable.

## Statistics

Descriptive statistics on covariates was applied, stratified on the RWD and non-RWD groups. Chi^2^-test or t-test for categorical and continuous variables, respectively was performed.

In order to qualify the discussion of potential sources of selection bias, non-response analyses were conducted. Chi^2^-test was used to test for differences between gender and self-assessed SES.

A logistic regression model was used to analyse the association between RWD and self-rated health. Three models were applied: Model 1 investigated the crude association; Model 2 investigated how loneliness and perceived stress, as indicators for mental health, mediated the association between RWD and self-rated health; Model 3 was a repetition of Model 2 with further adjustments by the addition of gender, age, self-assessed SES and negative childhood events. All analyses were adjusted for cluster on school-level using the “cluster” option in the STATA procedure “logistic”, which ensures robust Standard Errors despite the cluster effect. Results were reported as odds ratios (OR) with corresponding 95% confidence intervals (CI).

The software package STATA version 15.1 was used for the analyses.

### Ethics

The FOCA cohort was approved by the Danish Data Protection Agency (no. 1–16–02-461-16). The pupils gained access to the questionnaire through their UNI-login, a personal login given to all pupils in Denmark. It was voluntary for the adolescents to answer the questionnaire, and they had the right to withdraw their undertaking of participation at any time. All the answers were treated strictly confidential, and the adolescents were guaranteed full anonymity.

## Results

Distribution of self-rated health and baseline characteristics of the study population are presented in Table 1. The baseline characteristics were significantly different between the RWD and non-RWD groups except for gender (Table 1). Self-rated health was lower among pupils having RWD compared to pupils without RWD (*p* < 0.001).

**Table 1:** Characteristics of the study population

	Total *N* = 9748 *n* (%) or mean (SD)	RWD *N* = 953 (9.8) *n* (%) or mean (SD)	Non-RWD *N* = 8795 (90.2) *n* (%) or mean (SD)	p
Self-rated health				< 0.001^b^*
Low (%)	2606 (26.7)	338 (35.5)	2268 (25.8)	
High (%)	6386 (65.5)	494 (51.8)	5892 (67.0)	
Missing (%)	756 (7.8)	121 (12.7)	635 (7.2)	
Gender				0.055 ^b^
Female (%)	4973 (51.0)	458 (48.1)	4515 (51.3)	
Male (%)	4775 (49.0)	495 (51.9)	4280 (48.7)	
Alder, mean (SD)	15.8 (0.4)	16.0 (0.5)	15.8 (0.4)	< 0.001^c^*
Self-assessed SES				< 0.001^b^*
Low (%)	233 (2.4)	49 (5.1)	184 (2.1)	
Medium (%)	5411 (55.5)	552 (57.9)	4859 (55.3)	
High (%)	3529 (36.2)	248 (26.0)	3281 (37.3)	
Missing (%)	575 (5.9)	104 (10.9)	471 (5.4)	
Negative childhood events				< 0.001^b^*
0 events (%)	940 (9.6)	84 (8.8)	856 (9.7)	
1–3 events (%)	5769 (59.2)	433 (45.4)	5336 (60.7)	
4–7 events (%)	1616 (16.6)	181 (19.0)	1435 (16.3)	
8–11 events (%)	234 (2.4)	56 (5.9)	178 (2.0)	
Missing (%)	1189 (12.2)	199 (20.9)	990 (11.3)	
Loneliness				< 0.001^b^*
Not lonely (%)	7358 (75.5)	609 (63.9)	6749 (76.7)	
Lonely (%)	2379 (24.4)	341 (35.8)	2038 (23.2)	
Missing (%)	11 (0.1)	3 (0.3)	8 (0.1)	
Perceived stress ^a^, mean (SD)	14.3 (6.4)	16.5 (6.1)	14.1 (6.4)	< 0.001^c^*

^a^ = Scale from 0 to 40; higher = more stress, ^b^ = chi^2^, ^c^ = t-test, *statistical significant *p* < 0.05

Characteristics of the respondents, who did or did not answer the item concerning RWD are shown in Table 2. More boys (57.1%) than girls (42.9%) did not answer the RWD item (*p* < 0.001). No differences in self-assessed SES were observed.

**Table 2:** Non-response analyses in relation to the RWD question

	Total *N* = 10,200 *n* (%)	Response on RWD *n* = 9748 *n* (%)	Non-response on RWD *n* = 452 *n* (%)	p
Gender				0.001^a^*
Female (%)	5167 (50.7)	4973 (51.0)	194 (42.9)	
Male (%)	5033 (49.3)	4775 (49.0)	258 (57.1)	
Self-assessed SES				0.916^a^
Low SES (%)	241 (2.4)	233 (2.4)	8 (1.8)	
Medium SES (%)	5574 (54.7)	5411 (55.5)	163 (36.1)	
High SES (%)	3633 (35.6)	3529 (36.2)	104 (23.0)	
Missing (%)	752 (7.4)	575 (5.9)	177 (39.2)	

^a^ = chi^2^, *statistical significant *p* < 0.05

The association between RWD and self-rated health is shown in Table 3. RWD and self-rated health were significantly associated in all three models (Table 3). Adjustments for loneliness and perceived stress in Model 2 led to a reduction of the association between RWD and low self-rated health from 1.77 (95% CI: 1.51–2.09) in Model 1 to 1.47 (95% CI: 1.23–1.74) in Model 2. In the fully adjusted Model 3 the OR was 1.37 (1.14–1.66).

**Table 3:** Crude and adjusted OR (95% CI) for poor self-rated health

	Model 1		Model 2^a^		Model 3^b^	
	Crude *N* = 8992		Adjusted *N* = 8669		Adjusted *N* = 7959	
	OR	95% CI	OR	95% CI	OR	95% CI
RWD						
No	1		1		1	
Yes	1.77**	1.51–2.09	1.47**	1.23–1.74	1.37*	1.14–1.66
Loneliness						
Not lonely			1		1	
Lonely			1.53**	1.37–1.71	1.51**	1.34–1.69
Perceived stress			1.11**	1.10–1.12	1.11**	1.10–1.12
Gender						
Female					1	
Male					1.12	1.00–1.25
Age					1.17*	1.02–1.33
Self-assessed SES						
Low					2.02**	1.44–2.83
Medium					1.61**	1.43–1.80
High					1	
Negative childhood events						
0 events					1	
1–3 events					1.31*	1.08–1.59
4–7 events					1.57**	1.26–1.97
8–11 events					2.15**	1.49–3.09

^a^Adjusted for loneliness and perceived stress, ^b^adjusted for loneliness, perceived stress, gender, age, self-assessed SES and negative childhood events

*p < 0.05, **p < 0.001

## Discussion

### Main findings

A significant association between RWD and low self-rated health was found among Danish 9th grade pupils. Loneliness and perceived stress, as indicators of mental health, only explained a minor part of the association between RWD and self-rated health.

### Interpretation of findings

The association between RWD and self-rated health seems to be unexplored among adolescents [31–45]. Among adults, Moon et al. investigated the relationship between functional literacy/numeracy and self-assessed health and found both to be significantly associated even after controlling for covariates [32], which is in line with the findings of this study.

According to Zullig et al., adolescents mainly base their self-rated health on their mental health perceptions instead of their physical health [10]. Therefore, loneliness and perceived stress, as proxies for mental health, were expected to explain a major part of the association between RWD and self-rated health. However, the attenuation of the association between RWD and self-rated health after controlling for loneliness and perceived stress was minor. According to Breidablik et al. and Vingilis et al., self-rated health among adolescents is a multifactorial composite related to both physical health and non-physical health factors such as medical, psychological, social and lifestyle factors [9, 60]. This may explain why loneliness and perceived stress, as proxies for mental health, explained only a minor part of the association between RWD and self-rated health. Further adjustments in Model 3, did not alter the OR substantially, showing the rest of the covariates having a minor explanatory part in the association. The attenuation driven primarily by loneliness and perceived stress was in line with the argument put forward by Breidablik et al. and Vingilis et al. that self-rated health is a multifactorial composite [9, 60]. Future research should dive into self-rated health among adolescents to further understand the proposed multifactorial composite and their relationships.

### Strengths and limitations

This study was based on self-reported data, which might lead to inaccuracies. However, priority was given to include instruments validated in a Danish setting; SF-36, 10-Item Perceived Stress Scale and MacArthur Scale of Subjective Social Status, which therefore limited misclassifications in the present study. In Denmark, pupils with RWD often have a program on their computer that can read aloud text to speech. As it is expected that the pupils had the opportunity to have the questions read aloud from the computer when answering the questionnaire these inaccuracies are also not expected to be related to RWD. Therefore, the potential misclassifications are expected to be non-differentiated with associations between RWD and self-rated health approaching the null-hypothesis.

Regarding the self-reported measure of RWD, there is a potential risk of differentiated misclassification, as the item has not been validated. However, the aim was not to differentiate between pupils with and without dyslexia, but to differentiate pupils affected by RWD from those without. Future studies should validate the item used to identify pupils with RWD. Furthermore, the dichotomisation of the loneliness variable was based on studies looking at older people [56, 57]. This is not ideal but we were not able to find studies among adolescents using this variable. Therefore, there is a risk of misclassification with regard to this variable, but this is expected to be non-differentiated.

Of the 10,200 respondents eligible for this study, 452 were excluded as they did not answer the RWD question. The non-response analysis showed more girls than boys answered the RWD question. RWD and self-rated health are both associated with gender, whereas the prevalence of RWD is higher among boys, and girls tend to have a slightly lower self-rated health than boys [9, 61]. This may lead to underestimated associations between RWD and self-rated health. Self-assessed SES was not significantly associated with non-response, however missing values were present. Sensitivity analyses were performed, having responders and non-responders to RWD with missing values in self-assessed SES allocated to low and high self-assessed SES, respectively (1.36 (95% CI 1.15–1.61), data not shown, Additional file 1). This was repeated but allocating low self-assessed SES to non-responders and high self-assessed SES to RWD responders (1.38 (95% CI 1.16–1.63), data not shown, Additional file 1). Both extreme scenarios did not alter the OR in Model 3, and we are confident that the self-assessed SES did not cause selection bias.

The data was gathered on school-level which could introduce confounding by cluster. The consequence of RWD is expected to be very dependent of the school-environment. If the pupil is attending a school with a very supportive school-environment RWD is not expected to have the same impact, as it would have in a school with a very unsupportive environment. Therefore, we made cluster adjustments on school-level, which further strengthens the internal validity.

Despite that the cohort profile on FOCA concludes that it resembles the Danish background population [46], the prevalence of RWD are lower in this study than reported in the general population; According to the National Board of Social Services and the Patient’s Handbook, respectively, the prevalence of dyslexia in Denmark is estimated to be between 7 and 20% [27, 28]. Dyslexia is one branch of RWD, whereas the prevalence of RWD in itself is expected to be higher. The Programme for International Student Assessment (PISA) test conducted in 2015 found that 15% of Danish 15-year olds do not have sufficient functional reading skills [62]. In comparison, 9.8% of the sample in the present study was affected by RWD, possibly due to excluding pupils at schools for children with special needs.

This study is expected to have a reasonable internal validity, as the potential misclassification is expected to be non-differentiated and selection bias does not seem to threaten our results. It is therefore expected that the association between RWD and self-rated health may be even stronger than shown in the present study because of the low prevalence of RWD. The results may be generalized to adolescents attending public schools in Scandinavia.

## Conclusion

RWD and poor self-rated health was significantly associated in Danish adolescents. Perceived stress and loneliness explained a minor part of this association. The association between RWD and self-rated health may cause inequality in future health and the study findings underline the need to investigate the relationship in longitudinal studies. If support for a causal relationship can be found, it calls for early identification of RWD. Further studies are needed to understand which interventions may compensate RWD and potentially protect against health inequalities.

## Additional file

Additional file 1: Sensitivity analyses of self-assessed SES, Sensitivity analyses of self-assessed SES. (DOCX 31 kb)

## Abbreviations

CI: confidence interval
CPR: Civil Registration System
FOCA: Future Occupation of Children and Adolescents
HRQoL: health-related quality-of-life
OR: Odds ratio
PISA: Programme for International Student Assessment
RWD: reading and writing difficulties
SES: socioeconomic status
SF-36: the MOS 36-item Short Form
