# Supplementary material for: Reading and writing difficulties and self-rated health among Danish adolescents: cross-sectional study from the FOCA cohort
Source: BMC Public Health. 2019 May 10;19:537. doi: 10.1186/s12889-019-6931-x (PMC6511132; doi:10.1186/s12889-019-6931-x)
Supplement: Supplementary file 1 — Sensitivity analyses of self-assessed SES, Sensitivity analyses of self-assessed SES. (DOCX 31 kb) [file 12889_2019_6931_MOESM1_ESM.docx]

| Sensitivity analyses of self-assessed SES | | | | |
| --- | --- | --- | --- | --- |
|  | Total  N=10,200  n (%) | Response on RWD  N= 9748 (95.6)  n (%) | Non-RWD  N=452 (4.4)  n (%) | p |
| Self-assessed SES |  |  |  | <0.001^c^* |
| Low (%) | 241 (2.4) | 233 (2.4) | 8 (1.8) |  |
| Medium (%) | 5574 (54.7) | 5411 (55.5) | 163 (36.1) |  |
| High (%) | 3633 (35.6) | 3529 (36.2) | 104 (23.0) |  |
| Missing (%) | 752 (7.4) | 575 (5.9) | 177 (39.2) |  |
| Sens. analyses self-assessed SES test 1 ^a^ |  |  |  | <0.001^c^* |
| Low (%) | 816 (8.0) | 808 (8.3) | 8 (1.8) |  |
| Medium (%) | 5574 (54.7) | 5411 (55.5) | 163 (36.1) |  |
| High (%) | 3810 (37.4) | 3529 (36.2) | 281 (62.2) |  |
| Sens. analyses self-assessed SES test 2 ^b^ |  |  |  | <0.001^c^* |
| Low (%) | 418 (4.1) | 233 (2.4) | 185 (40.9) |  |
| Medium (%) | 5574 (54.7) | 5411 (55.5) | 163 (36.1) |  |
| High (%) | 4208 (41.3) | 4104 (42.1) | 104 (23.0) |  |

^a^= Missing SES and response on RWD => low SES. Missing SES and non-response RWD => high SES, ^b^= Missing SES and response on RWD => high SES. Missing SES and non-response RWD => low SES, ^c^= chi^2^, *statistical significant p<0.05

| Model 3, adjusted logistic regression analyses of the association between RWD and low self-rated health using self-assessed SES test 1 and self-assessed SES test 2 | | | | | | |
| --- | --- | --- | --- | --- | --- | --- |
|  | Model 3 | | Model 3^a^ | | Model 3^b^ | |
|  | Adjusted  N=7959 | | Adjusted  N=8361 | | Adjusted  N=8361 | |
|  | OR | 95% CI | OR | 95% CI | OR | 95% CI |
| RWD |  |  |  |  |  |  |
| No | 1 |  | 1 |  | 1 |  |
| Yes | 1.37* | 1.14-1.66 | 1.36** | 1.15-1.61 | 1.38** | 1.16-1.63 |
| Loneliness |  |  |  |  |  |  |
| Not lonely | 1 |  | 1 |  | 1 |  |
| Lonely | 1.51** | 1.34-1.69 | 1.53** | 1.36-1.73 | 1.52** | 1.35-1.72 |
| Perceived stress | 1.11** | 1.10-1.12 | 1.11** | 1.10-1.12 | 1.11** | 1.09-1.12 |
| Gender |  |  |  |  |  |  |
| Female | 1 |  | 1 |  | 1 |  |
| Male | 1.12 | 1.00-1.25 | 1.09 | 0.98-1.21 | 1.10 | 0.98-1.22 |
| Age | 1.17* | 1.02-1.33 | 1.16* | 1.02-1.31 | 1.16* | 1.03-1.32 |
| Self-assessed SES |  |  |  |  |  |  |
| Low | 2.02** | 1.44-2.83 | 1.57** | 1.28-1.93 | 1.94** | 1.40-2.68 |
| Medium | 1.61** | 1.43-1.80 | 1.61** | 1.44-1.80 | 1.54** | 1.38-1.72 |
| High | 1 |  | 1 |  | 1 |  |
| Negative childhood events |  |  |  |  |  |  |
| 0 events | 1 |  | 1 |  | 1 |  |
| 1-3 events | 1.31* | 1.08-1.59 | 1.28* | 1.07-1.53 | 1.27* | 1.06-1.52 |
| 4-7 events | 1.57** | 1.26-1.97 | 1.58** | 1.29-1.94 | 1.56** | 1.27-1.91 |
| 8-11 events | 2.15** | 1.49-3.09 | 2.05** | 1.47-2.86 | 2.15** | 1.54-3.00 |

^a^ Using self-assessed SES test 1, ^b^ Using self-assessed SES test 2

* p <0.05, **p<0.001
